# Family planning and multiple sclerosis – part 2: Does real-world practice align with Swedish treatment guidelines?

**DOI:** 10.1177/20552173261472249

**Published:** 2026-07-24

**Authors:** Alejandra Machado, Emma Pettersson, Fitsum Sebsibe Teni, Emilie Friberg, Katharina Fink

**Affiliations:** 1 27106 Division of Insurance Medicine, Department of Clinical Neuroscience, Stockholm, Sweden; 2 27106 Division of Neuro, Department of Clinical Neuroscience, Stockholm, Sweden

**Keywords:** Multiple sclerosis, disease-modifying therapies, family planning, insights, guidelines

## Abstract

**Background and objectives:**

Managing family planning and treatment in women with multiple sclerosis (MS) requires balancing disease control with medication safety. Despite guidelines, practice often adapts to individual needs, with clinicians adjusting disease-modifying therapies (DMTs) to protect maternal and fetal health. This study examines pregnancy-related MS treatment in Sweden.

**Methods:**

We identified 130 women (153 pregnancies) from a 2021 online MS questionnaire and linked responses to the Swedish MS register to capture DMT use during three periods: 25 weeks before conception, pregnancy (≈40 weeks), and 25 weeks postpartum. DMTs were classified as “accepted,” “discontinuation recommended”—requiring monitoring, or “not recommended” in planned pregnancy.

**Results:**

Before conception, among 153 pregnancies, 26% involved DMTs considered acceptable before and during pregnancy, while 55% involved DMTs for which discontinuation before conception is recommended—of which rituximab accounted for nearly half of all pregnancies (47%, n = 72). Three percent were exposed to ´not recommended´ DMTs, and 16% had no prior treatment. Overall, 29% discontinued DMTs before or shortly after conception, while 58% (n = 88) had ongoing or recent treatment exposure, defined as treatment administered within 6 months before conception or, in a minority of cases, after conception. Among these, 50% (n = 77) involved rituximab—and 13% remained untreated. Postpartum, 58% resumed treatment, 3% remained under long-lasting DMT effects, and 39% did not initiate DMT, likely due to breastfeeding.

**Discussion:**

Overall, real-world practice in Sweden broadly aligns with national recommendations, with widespread use of rituximab before pregnancy reflecting its perceived safety and durable effect, alongside some individualized deviations from discontinuation guidance.

## Introduction

Multiple sclerosis (MS) is often diagnosed in women of childbearing age, influencing family planning decisions.^1^ Advances in disease-modifying treatments (DMTs) have improved disease control, allowing more women with MS to consider pregnancy. However, limited safety data during pregnancy and lactation complicate treatment choices.^2^ While pharmaceutical labels often advise against DMT use, real-world evidence suggests that some therapies may be safe for use during pregnancy.^3^ The risk-benefit balance must be reassessed at each stage of pregnancy.^2^

Ideally, disease stability should be achieved 6–12 months before conception, although unplanned pregnancies are common.^4^ Swedish national and MS society guidelines recommend discontinuing most DMTs before conception, with timing depending on the specific drug, but treatment may be continued in some cases to prevent rebound activity.^5,6^ In contrast, international guidelines and pharmaceutical labels are more conservative, often advising avoidance during pregnancy due to limited safety data. This discrepancy contributes to variations between recommendations and real-world practice, where treatment decisions are individualized to balance maternal disease control and fetal safety.

Teriflunomide and fingolimod are contraindicated during pregnancy due to teratogenic risks.^7^ In contrast, glatiramer acetate and interferons are considered safe.^3,8^ Natalizumab may be continued until late in pregnancy or switched to a high-efficacy therapy to reduce the risk of rebound.^2^ Rituximab, although used off-label, is widely used in Sweden due to its perceived safety profile and long-lasting effects, making it a preferred option before pregnancy.^9,10^ It has a long-lasting effect, extends beyond its half-life, providing continued disease control even after discontinuation and offering protection during pregnancy and the postpartum period.^11^ Consequently, it is often used when previous DMTs are incompatible with pregnancy.^12^

This study aims to assess the treatment strategies across pregnancy stages in Sweden and how these align with current national recommendations.

## Materials and methods

### Study population and data sources

We analyzed 153 pregnancies from 130 women, selected from respondents to a previous survey (May–Sept 2021) of women aged 20–50 in the Swedish MS Register (SMSreg) who responded a pregnancy-related question (*see Part 1 of this study*). These 130 women were selected based on their available registered data regarding estimated birth dates (between 2009-12-14 and 2021-11-13). To assess treatment patterns across pregnancy stages, we defined three periods: (1) a pre-conception period of 25 weeks (equivalent to 6 months) to the estimated conception date, (2) the pregnancy period (40 weeks), and (3) a postpartum period of 25 weeks following the estimated delivery date. The estimated conception date (“start of pregnancy”) was calculated by subtracting 40 weeks from the estimated delivery date recorded in the registry, which was entered by the physician based on patient report when the pregnancy was confirmed. Maternal age at delivery was calculated from date of birth and estimated delivery date.

Statistics Sweden linked pseudo-anonymized survey responses to clinical data from the SMSreg (up to 2021), including MS type, time since diagnosis, latest DMT and dose dates, EDSS-based severity (mild, moderate, severe)—was included only if within 3 years prior to the survey response; otherwise set to missing), and pregnancy details (estimated delivery date, breastfeeding). Women's age at estimated date of delivery was calculated from birth date. Other sociodemographic information such as education, country of birth, type of living area, etc., were linked from the longitudinal integrated database for health insurance and labor market studies (LISA) as of 31 December 2019.

For these 153 observations, DMTs were reclassified by safety level based on the current recommendations from the Swedish MS society for women of childbearing age who desire pregnancy or are not on reliable birth control.^5,6^ The categories are described as follows:

1. “*Accepted” exposure during conception and pregnancy:* a) Seen as safe: interferons and glatiramer acetate; b) Continuous treatment during pregnancy until week 30 needed due to risk of rebound: natalizumab.
2. “*Discontinuation recommended” before or at conception due to unclear or plausible risk:* Dimethyl fumarate (DMF) at conception, Anti-CD20 monoclonal antibodies such as rituximab, ocrelizumab, and ofatumumab – with a washout period of 3–4 months before conception. Alemtuzumab – with a washout of 4 months, and Cladribine with a 6-month washout.
3. “*Not recommended”* DMTs with known teratogenic potential where prescription or initiation before a desired or planned pregnancy should be avoided: teriflunomide, fingolimod, and hematopoietic stem cell transplantation (HSCT).
4. *No DMT treatment*

### Statistical analysis

Descriptive statistics were used to summarize the demographic and clinical characteristics of the sample. Descriptive statistics was also used to determine the types of DMTs used before and during pregnancy as well as postpartum among the women in the study. Statistical analyses were performed using R software (version 4.5.0).

## Results

Among the 130 women, 107 (82.3%) had a single pregnancy, while 23 (17.7%) contributed two pregnancies. The mean age at delivery was 33 years (standard deviation (SD) of 4.3 years). The median estimated delivery date was April 22, 2018. At the time of survey response, almost all women had relapsing-remitting MS (98.5%) and mild disability (EDSS=0–2.5; 87%). Additional cohort characteristics are presented in Table 1.

**Table 1.** Cohort demographics and clinical characteristics at the time of the survey (or in 2019*).

Characteristic	N = 130
**Age at estimated date of delivery (years)**	
Mean (SD)	33 (4.3)
Born in Sweden*, n (%)	118 (90.8%)
Type of living area*, n (%)	
City	71 (54.6%)
town/suburb	36 (27.7%)
rural area	23 (17.7%)
Educational level*, n (%)	
Primary	5 (3.9%)
high school	33 (25.4%)
university/college	92 (70.8%)
Civil status*, n (%)	
Single/not registered partnership	67 (51.5%)
married/registered partnership	63 (48.5%)
Children under 20 years living at home*, n (%)	
No	33 (25.4%)
Yes	97 (74.6%)
**MS phenotype**	
Relapsing-remitting MS (RRMS)	128 (98.5%)
Progressive MS	<5 (1.6%)
**EDSS categorization, n (%)**	
Mild (0–2,5)	107 (82.3%)
Moderate (3–5,5) or Severe (≥6)	16 (12.3%)
Unknown	7 (5.4%)
**Time since diagnosis (years)**	
Mean (SD)	9.06 (4.3)
Median (IQR)	9.00 (6.00–11.00)

*Abbreviations*: N/n, sample size; SD, standard deviation; MS, multiple sclerosis; IQR Interquartile range

**Figure 1.**
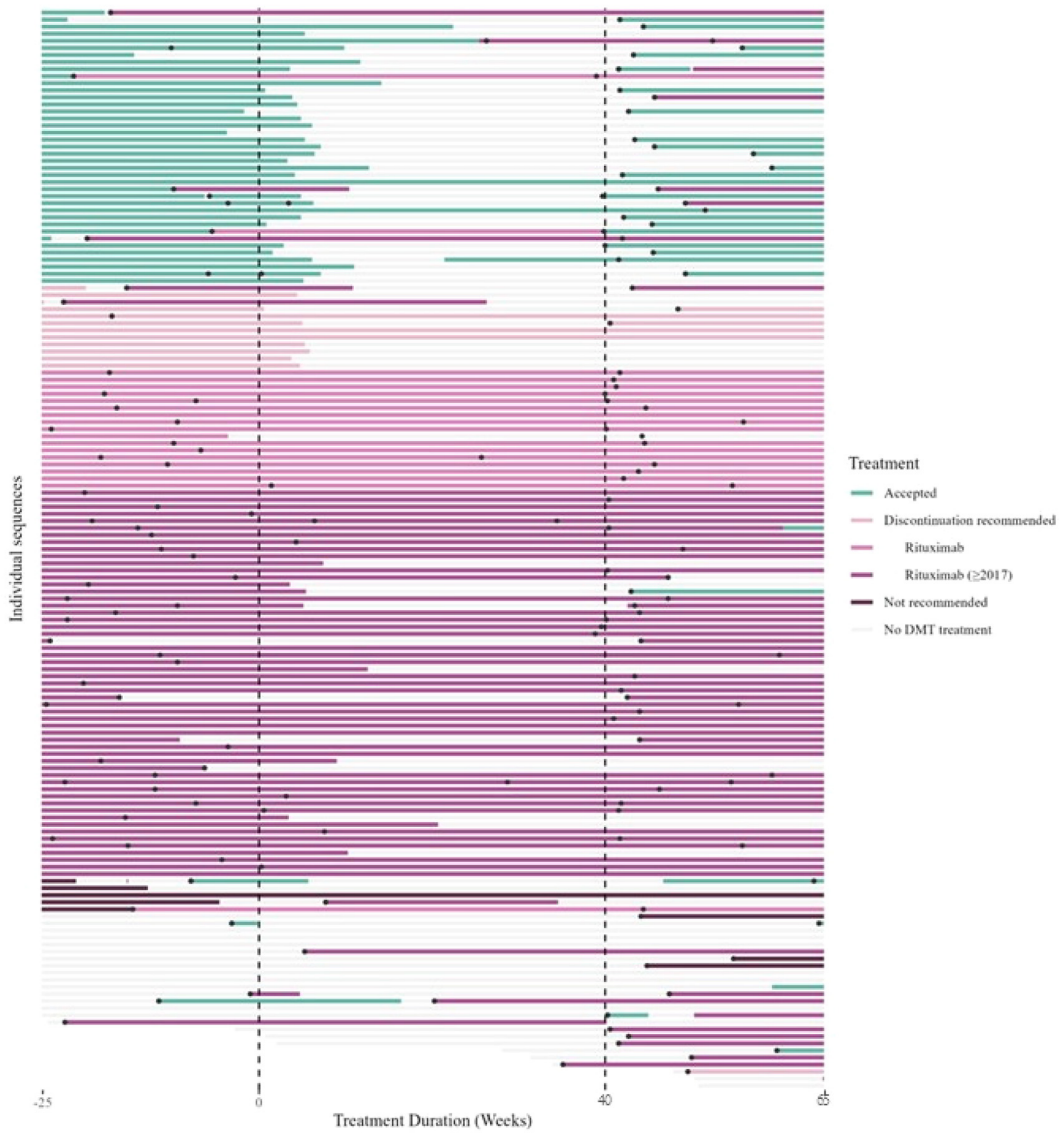
Prescribed DMT treatments of 153 pregnancies before, during, and after pregnancy over a 90-week period. The discontinuation lines indicate the start (week 0) and end (week 40) of the estimated pregnancy period, as well as 25 weeks before (−25) and after (65) pregnancy. Each line represents the treatment sequence for an individual pregnancy, providing a visual representation of the changes in treatment over time. Black spots represent the last DMT exposure (registered dose date) prior or after conception (discontinued line in week 0) or estimated delivery (discontinued line in ≥ week 40). Treatments are color-coded as follows: *Accepted* (green); *Discontinuation recommended* (all three pink tones: light, medium and dark), *Not recommended* (plum), and *No DMT treatment* (gray). Rituximab before (medium pink) or after 2017 (dark pink) is depicted separately from the other antiCD20 (light pink) due to its high percentage of use. Further, the split of rituximab before and after 2017, accounts for different procedures to register the data (e.g., not to modify stop date if the patient remains under the drug's long-lasting effect).

Treatment patterns differed across the pre-conception, pregnancy, and postpartum periods (Figure 1). Prior to conception, 26% of pregnancies (n = 39/153) involved DMTs considered acceptable before and during pregnancy, while 55% (n = 84) involved DMTs for which discontinuation before conception is recommended, of which rituximab accounted for nearly half (47%, n = 72). Only 3% (n = 5) involved DMTs not recommended for pregnancy, while 16% (n = 25) had no treatment.

During the 6-month pre-conception period, 29% (n = 45/153) discontinued treatment before or shortly after the estimated conception date, while 58% (n = 88) continued treatment into pregnancy. Of these 88 pregnancies, 73 continued the same treatment, whereas 10 involved a treatment switch, either to rituximab (RTX, n = 9) or to an accepted DMT (n = 1). In addition, five pregnancies initiated treatment during this period (RTX, n = 3; accepted DMT, n = 2). The remaining 13% (n = 20) had not received any treatment prior to conception.

Among pregnancies with DMT exposure (58%, n = 88/153), the majority reflected treatment administered prior to conception with a continuing biological effect during pregnancy (n = 73/88). Rituximab was the most common DMT during pre-conception (74%, n = 65/88), while fewer pregnancies involved other DMTs classified as accepted (n = 4) or discontinuation recommended (n = 3). Only one pregnancy was classified as “not recommended” due to prior hematopoietic stem cell transplantation (HSCT), although the procedure had been performed 2 years before conception, a timeframe that may not necessarily contraindicate pregnancy.

Notably, among the 77 pregnancies involving rituximab (65 ongoing, nine switched, and three initiated), treatment was classified by the treating neurologist as continuing into or throughout pregnancy, reflecting the prolonged biological effect of these therapies rather than active dosing during pregnancy. Specifically, 28 women received their last rituximab dose 3–6 months before conception, whereas 23 received it within 3 months before conception. A smaller subset of women received DMT after the estimated conception date, most likely due to unrecognized pregnancy rather than planned exposure (Figure 1). In the postpartum period, treatment resumption patterns varied substantially: The majority of pregnancies (61%, n = 93) were exposed to treatment postpartum, either through treatment resumption/initiation after birth (58%, n = 88) or presumed continuation of treatment effects (3%, n = 5). On the contrary, 39% of the pregnancies (n = 60) did not resume or initiate treatment (Figure 1).

## Discussion

This study explored treatment strategies among a subsample of women with MS who participated in a 2021 survey in Sweden and had one or two pregnancies between 2009 and 2021. Rituximab was the most used drug, with over half of the pregnancies already on rituximab or switching to it prior to conception. Very few women received RTX after conception – likely reflecting unrecognized pregnancy at the time of administration. This finding underscores its widespread use in Sweden, even among women of childbearing age.^9,10^ This contrasts sharply with practices in other European countries, where prior to conception, women of childbearing age were more often treated with other non-high-efficacy drugs (53%–64%) than with similar high-efficacy drugs (23%–24%).^13,14^ In an Italian study by Moccia et al.,^14^ none of the 154 pregnancies were exposed to rituximab or initiated it for family planning. Similarly, in the German study by Bast et al.,^13^ only 5% of the 3752 pregnancies involved anti-CD20 therapy prior to pregnancy.

Among the pregnancies exposed to DMTs within the 6 months preceding conception or shortly thereafter, most were likely to remain protected during pregnancy due to the drug's prolonged effect. Exposure to rituximab within 6 months prior to conception, and occasionally shortly after, reflects the common practice of maintaining protection against disease activity during pregnancy and postpartum, owing to its long-lasting effect exceeding its washout.^11^ This approach is also used when previous treatments are contraindicated during pregnancy.^12^ Despite rituximab being an off-label therapy for MS,^9,10^ its use is endorsed by national recommendations.^5,6^ Our findings align with the recommendations from the professionals’ Swedish MS Society, suggesting that these strategies are deliberate and increasingly implemented in clinical practice, a strategy that may have become even more common in recent years.

This study has several limitations that should be considered. First, detailed measures of inflammatory disease activity, such as annualized relapse rate (ARR) and MRI activity, were not consistently available in the registry, limiting our ability to assess whether treatment patterns were appropriately tailored to disease activity. Additionally, the relatively small sample size and inconsistencies in registry data, particularly the use of estimated rather than actual birth dates to determine pregnancy timing, may affect the precision of our findings. Despite these limitations, the study provides valuable exploratory insights into treatment strategies in this population. Future research with larger cohorts, longer follow-up periods, and more comprehensive data is needed to confirm whether real-world practices align with national recommendations and to better understand evolving trends in family planning and MS treatment management in Sweden.

Overall, our findings suggest that real-world clinical practice in Sweden largely aligns with national recommendations, particularly regarding the strategic use of rituximab prior to conception to maintain disease control during pregnancy. However, deviations from formal discontinuation recommendations highlight the role of individualized treatment decisions in clinical practice.
